# ALDH2 Deficiency Promotes Mammary Epithelial Stemness and Proliferative Morphogenesis Through Oxidative Stress, RANKL Induction, and Estrogen Receptor Signaling

**DOI:** 10.3390/cells15181632

**Published:** 2026-09-09

**Authors:** Zhikun Ma, Amanda B. Parris, Miles Lester, De’ja Gissendanner, Vasilis Vasiliou, Xiaohe Yang

**Affiliations:** 1 Department of Biological and Biomedical Sciences, Julius L. Chambers Biomedical/Biotechnology Research Institute, North Carolina Research Campus, North Carolina Central University, 500 Laureate Way, Kannapolis, NC 28081, USA; zma@nccu.edu (Z.M.); ablack18@nccu.edu (A.B.P.);; 2 Department of Environmental Health Sciences, Yale School of Public Health, Yale University, New Haven, CT 06520, USA; vasilis.vasiliou@yale.edu

**Keywords:** aldehyde dehydrogenase 2, oxidative stress, mammary epithelial cell repopulation, estrogen receptor, RANKL, cyclin D1

## Abstract

Alcohol consumption is associated with increased breast cancer risk, partly due to the accumulation of toxic aldehydes like acetaldehyde, a carcinogenic byproduct of ethanol metabolism. Acetaldehyde Dehydrogenase 2 (ALDH2), a key mitochondrial enzyme, detoxifies acetaldehyde and other reactive aldehydes that drive oxidative stress, DNA damage, and hormonal dysregulation—processes central to carcinogenesis. Although alcohol consumption has been implicated in breast cancer, the role of ALDH2 deficiency itself, in the absence of exogenous alcohol exposure, in mammary gland biology and cancer susceptibility remains unclear. Genetic variants that impair ALDH2 activity are highly prevalent in East Asian populations, where carriers of inactive ALDH2 alleles exhibit impaired aldehyde detoxification. While such individuals are more susceptible to alcohol-related cancers, the effects of ALDH2 deficiency on mammary gland development and homeostasis without alcohol exposure remain unexplored. To investigate the effects of ALDH2 deficiency on mammary proliferation and development, we utilized a C57BL/6-based ALDH2 knockout (Aldh2−/−) mouse model. Our findings revealed that Aldh2−/− mice displayed hyperproliferative mammary glands with increased epithelial cell density, ductal expansion, and increased numbers of Ki67+ cells. Flow cytometry analysis revealed expansion of luminal and basal epithelial subpopulations, accompanied by enhanced mammary epithelial stemness, as indicated by increased mammosphere formation and colony-forming efficiency. At the molecular level, ALDH2 deficiency activated oxidative stress pathways, reflected by elevated 8-OHdG, p38 MAPK, NF-κB, and Nrf2 signaling, along with DNA damage responses involving p53 and H2A.X. We also identified a novel upregulation of RANK and RANKL in Aldh2−/− mammary glands, identifying the RANK/RANKL upregulation associated with NF-κB/p38 MAPK activation and enhanced mammary stemness. Furthermore, hormonal dysregulation was observed, with a significant increase in ERα and PR expression and phosphorylation. Dysregulated ER signaling correlated with enhanced erbB3 activation and downstream signaling, including the cyclin D1–pRb-E2F1 axis. These findings suggest that ALDH2 deficiency, possibly through accumulated endogenous aldehydes, profoundly alters mammary morphogenesis, epithelial repopulation, and stemness. These effects are associated with activation of oxidative stress and DNA damage pathways, together with upregulation of RANKL, estrogen receptor and receptor tyrosine kinase signaling. This study is the first to identify ALDH2 deficiency as a novel factor associated with mammary epithelial alterations that may create a tissue state that could predispose to oncogenic transformation.

## 1. Introduction

Aldehyde dehydrogenase 2 (ALDH2) plays a critical role in the detoxification of aldehydes, particularly acetaldehyde, and is essential for maintaining cellular homeostasis [1]. Beyond alcohol metabolism and environmental toxin clearance, ALDH2 also removes endogenous aldehydes generated through amino acid metabolism, alcohol-related intermediates, and lipid peroxidation [2,3]. Understanding the impact of ALDH2 function in regulating endogenous aldehydes across different tissues is therefore critical.

The ALDH2 polymorphism, particularly the ALDH2*2 (rs671) allele, is associated with a marked reduction in ALDH2 enzyme activity [4]. This polymorphism is highly prevalent in East Asian populations, with an estimated allele frequency of 30–50% [5]. Epidemiological studies indicate that ALDH2 polymorphisms significantly influence susceptibility to multiple diseases, including cancers [1,6]. Increasing evidence suggests that the functional deficiency caused by ALDH2 polymorphisms and the resulting elevated aldehyde levels may promote the development and progression of esophageal, head and neck, liver, and colorectal cancers [7,8,9,10]. The relationship between ALDH2 polymorphisms and breast cancer, however, remains unclear. While some studies reported increased breast cancer risk associated with ALDH2 polymorphisms in certain populations [11,12], other research finds no significant association [13,14], highlighting the need for further investigation. Studies using preclinical models with well-controlled experimental conditions are particularly important to advance understanding of these critical issues, yet studies in this area remain scarce.

The biochemical functions of ALDH2, including its roles in mitochondrial activity, oxidative stress regulation, and DNA damage response, have been extensively studied. In particular, biochemical studies demonstrate that ALDH2 mitigates oxidative stress associated with cellular metabolism [15,16]. As a mitochondrial enzyme, ALDH2 aids in the detoxification of harmful aldehydes generated during metabolic processes [17]. ALDH2 deficiency increases oxidative stress in cells and has been associated with various pathological conditions, including cardiovascular diseases and alcohol-related pathologies [18,19,20,21]. Elucidating the diverse roles of ALDH2 in cellular metabolism and its molecular interactions with oxidative stress pathways is therefore critical for developing potential interventions against oxidative stress-related diseases. However, most research has focused on the liver, cardiovascular and other tissues [16,18,22,23], with limited evidence regarding the impact of ALDH2 deficiency on mammary gland physiology. Aldh2−/− mouse models have been useful in defining ALDH2’s in vivo roles in organs such as the liver and the brain [22,24,25,26], but its role in mammary development and the underlying mechanisms are yet to be explored. Although both models involve impaired ALDH2 function, complete genetic deletion of Aldh2 in mice is mechanistically distinct from the human ALDH2*2 (rs671) polymorphism. The Aldh2−/− model results in complete loss of ALDH2 expression, whereas the ALDH2*2 variant encodes an inactive subunit that exerts a dominant-negative effect on the tetrameric ALDH2 enzyme. The ALDH2*2 variant exerts a dominant-negative effect on the homotetrameric enzyme, reducing residual activity to ~13–17% of wild-type levels in heterozygotes (ALDH2*1/2) and to virtually zero in homozygotes (ALDH2*2/2) [27,28,29]. Thus, the Aldh2−/− model provides a proof-of-concept system for investigating the biological consequences of ALDH2 loss of function under controlled experimental conditions. Allelic mutant models will be important for defining effects specific to the human ALDH2*2 variant.

Mammary gland development is highly susceptible to various environmental and endogenous factors that alter cellular dynamics, particularly with respect to breast cancer risk [30]. Previous findings indicate that aberrations in mammary epithelial cell proliferation and differentiation often precede the onset of breast cancer [31]. Recent advances indicate that mammary stem cells, which are essential for mammary tissue development and differentiation, are particularly vulnerable to metabolic disruptions [32,33]. According to cancer stem cell theory, mutated or dysregulated mammary stem/progenitor cells may serve as the origin of cancer stem cells [34]. Altered stem/progenitor cell dynamics, reflected in mammary epithelial repopulation and functional changes in their stemness, are emerging biomarkers for assessing mammary proliferation and differentiation [35]. Considering the biochemical changes associated with ALDH2 deficiency observed in vitro [15,16], it is essential to examine its in vivo effects on mammary development and their implications for breast cancer risk.

In this study, we employed the C57BL/6-Aldh2−/− mouse model to investigate the biological consequences of ALDH2 deficiency in mammary tissues. Our findings demonstrate that ALDH2 knockout induces pronounced proliferative activity, morphological alterations, and dysregulated signaling pathways in mammary epithelial cells, creating a biological context that could predispose to oncogenic transformation. Central to these observations were robust changes in mammary epithelial cell repopulation dynamics, underscoring a critical role for ALDH2 in regulating epithelial stemness and progenitor cell function. Together, these findings provide new insight into how ALDH2 deficiency alters mammary development and epithelial homeostasis in ways that could facilitate subsequent oncogenic transformation.

## 2. Materials and Methods

### 2.1. Reagents

p-p38 MAPK (Thr180/Tyr182) (4511), p38 MAPK (9212), p-NF-κB (Ser536) (3033), p-NF-κB (8242), Nrf2 (12721), p-H2A.X (Ser139) (9718), H2A.X (2595), PR (8757), p-PR (Ser190) (3171), p-EGFR (Ser1046/1047) (2238), p-erbB-3 (Tyr1197) (4561), p-Akt (Ser473) (4060), p-Erk1/2 (Thr202/Tyr204) (9101), p-ERα (Ser118) (2511), c-Myc (5605), p-Rb (Ser807/811) (8516), Rb (9309), and GAPDH (5174) primary antibodies were obtained from Cell Signaling Technology (Danvers, MA, USA). Primary antibodies against ALDH2 (sc-100496), p53 (sc-126), MDM2 (sc-965), Bcl-2 (sc-7382), cyclin D1 (sc-246), Akt1 (sc-5298), Erk2 (sc-1647), ERα (sc-8002), ERβ (sc-8974), EGFR (sc-373746), erbB-3 (sc-285) and E2F1 (sc-251) were purchased from Santa Cruz Biotechnology (Dallas, TX, USA). RANK (MA5-16153) and RANKL (PA6-110266) were from Invitrogen (Carlsbad, CA, USA). HRP-conjugated anti-rabbit (7074) and anti-mouse (7076) secondary antibodies were from Cell Signaling Technologies. For immunohistochemistry, Ki67 (PA5-19462) antibody was purchased from Invitrogen, 8-OHdG (sc-66036) from Santa Cruz Biotechnology and the secondary antibody for immunohistochemistry was included in the VECTASTAIN Elite ABC kit (Vector Labs, Newark, CA, USA). Flow cytometry antibodies against CD16/32 (553141), CD49f (555735), CD24 (553260), and CD61 (553345) were purchased from BD Biosciences (San Diego, CA, USA), while those against CD31 (102508), CD45 (103106), and Ter-119 (116208) were obtained from BioLegend (San Diego, CA, USA).

### 2.2. Animals and Husbandry

Female C57BL/6 mice were obtained from Jackson Laboratory (Bar Harbor, ME, USA), while C57BL/6- Aldh2−/− mice were generously provided by Dr. Vasilis Vasiliou (Yale University). The mice were maintained on a standard diet in a temperature-controlled facility with a 12 h light–dark cycle. A total of 15 mice per group were used for various analyses, including whole-mount analysis, histopathology, Western blotting, RNA extraction, mammosphere and colony-forming cell (CFC) assays, and flow cytometry, as described in the respective assay protocols. Unless otherwise indicated, all experiments were performed using mammary tissues or cells obtained from 16-week-old mice. All experimental procedures were approved by the Institutional Animal Care and Use Committee.

### 2.3. Whole-Mount Analysis

The #4 inguinal mammary glands were dissected from control and Aldh2−/− mice at experimental endpoints and mounted on glass slides. Glands were fixed overnight in Carnoy’s solution, rehydrated through ethanol washes, stained with carmine alum, dehydrated with ethanol, cleared in xylene, and mounted with Permount, following established protocols [36]. Whole-mount images were captured using a Nikon microscope. The complexity of the mammary ductal trees was assessed by quantifying the number of lateral buds in an area of 10 mm^2^. Statistical significance was determined using Student’s *t*-test based on samples from five mice per group.

### 2.4. Histology and Immunohistochemistry

The mammary tissues were fixed in formalin and processed for paraffin embedding and tissue sectioning. For H&E staining, sections were stained with hematoxylin and eosin. For immunohistochemistry (IHC), the VECTASTAIN Elite ABC kit (Vector Laboratories) was used. Slides were incubated overnight at 4 °C with primary antibodies at appropriate dilutions (8-OHdG 1:300, p-ERα 1:100, and cyclin D1 1:100), followed by incubation with a biotinylated secondary antibody and ABC reagent. Diaminobenzidine (DAB) was used for color development, and slides were counterstained with hematoxylin [36]. For immunofluorescence staining, after primary antibody incubation (Ki67 1:1000) overnight in a humidity chamber at 4 °C, the sections were washed with PBS/0.1% Triton X100 buffer and incubated with the appropriate Alexa-Fluor-488 secondary antibodies at room temperature for 2 h. After mounting with an anti-fade mounting media with DAPI (Vector Laboratories), images were acquired using Nikon Eclipse 80i microscope.

### 2.5. MEC Isolation and Flow Cytometry

The #4 inguinal mammary glands from 16-week-old mice were collected and homogenized with a tissue chopper. The tissues were digested with collagenase and hyaluronidase for 2 h at 37 °C, as described in our previous reports [37]. The resulting organoids were further digested with trypsin and dispase/DNase I and filtered through a 40 µm mesh strainer. The single-cell suspension of mammary epithelial cells (MECs) was used for flow cytometry and functional assays. For flow cytometry analysis, 1 × 10^6^ primary mammary epithelial cells were stained with fluorescent antibodies against lineage and cell surface markers, as previously described [37]. Briefly, cells were first incubated with anti-CD16/CD32 to block Fc receptors. PE-conjugated anti-CD31, anti-TER-119, and anti-CD45 were used to exclude endothelial, erythroid, and leukocyte cells, respectively. Biotin–streptavidin–APC anti-CD24, biotin–streptavidin–APC anti-CD61, and FITC-conjugated anti-CD49f were then applied. The dead cells were excluded for subpopulation analysis by gating out 7-AAD positive cells. Lin- CD24/CD49f cells were used to identify stromal, luminal and basal subpopulations, whereas Lin- CD61/CD49f was used for the identification of progenitor-enriched cells, represented by the P2 subpopulation mentioned in Section 3.2. FlowJo v10 analysis software was used for gating and quantification of the individual cell populations.

### 2.6. Colony-Forming Cell Assay

Single cell suspensions of primary mammary epithelial cells were plated at a density of 4 × 10^3^ cells per 60 mm plate and cultured in a 5% CO_2_ atmosphere. Following a 10-day incubation period, colonies were rinsed with PBS, fixed with acetone:methanol (1:1), and stained with Wright’s Giemsa [37]. Images were acquired with the Nikon SMZ 745T microscope (Melville, NY, USA) using Nikon Elements Imaging System software. The colony numbers of the two groups were quantified and statistically analyzed.

### 2.7. Mammosphere Assay and 3D Culture

Isolated primary mammary epithelial cells were seeded at a density of 2.5 × 10^4^ cells per well in ultra-low attachment 24-well plates. Cells were cultured in EpiCult-B Mouse Medium (Stemcell Technologies, Vancouver, BC, Canada) supplemented with 10 µg/mL insulin (Sigma, Burlington, MA, USA), 1 µg/mL hydrocortisone (Sigma), 1× B-27 (Thermo Scientific, Waltham, MA, USA), 20 ng/mL EGF (Stemcell Technologies), 20 ng/mL basic fibroblast growth factor (Stemcell Technologies), 4 µg/mL heparin (Stemcell Technologies), and 50 µg/mL gentamicin for 7 days. Primary mammospheres > 30 μm were counted and imaged. For secondary sphere formation assays, primary mammospheres were dissociated with trypsin, and the resulting single-cell suspensions were replated at a density of 1 × 10^3^ cells per well under identical conditions to form secondary spheres. After an additional 7 days of incubation, secondary mammospheres were counted and imaged [37]. The data from primary and secondary mammosphere assays in triplicate were analyzed with Student’s *t*-test.

For the 3D culture assay, 1.5 × 10^4^ primary mammary epithelial cells were seeded in Matrigel in 48-well plates and cultured for 10 days. Subsequently, the colonies formed in the semi-solid medium were stained with crystal violet, quantified and imaged. The data from triplicate experiments were analyzed with Student’s *t*-test.

### 2.8. Western Blotting

Mammary tissues were homogenized in lysis buffer as described previously [37]. Protein concentrations of the lysates were measured with the BCA Protein Assay kit (Thermo Scientific). Equal amounts of protein (50 μg) were separated by SDS–PAGE, transferred to nitrocellulose membranes and blocked with 5% milk in Tris-buffered saline with Tween 20 (TBST). The membranes were incubated overnight at 4 °C with primary antibodies diluted in 5% bovine serum albumin (BSA) in TBST. After washing, the membranes were incubated with HRP-conjugated secondary antibodies for 1 h at room temperature, followed by detection with enhanced chemiluminescence reagents (Thermo Scientific) [36]. Protein bands were visualized with an Azure imaging system. Western blot band intensities were quantified by densitometry, background-corrected, and normalized to the corresponding loading controls. Phosphorylated proteins were further normalized to their respective total protein levels. Data from three biological samples per group are presented as fold change relative to the control group.

### 2.9. RNA Isolation and Quantitative Real-Time PCR

Mammary tissues from experimental groups were harvested and snap-frozen in liquid nitrogen. RNA was extracted using the RNeasy Mini Kit (Qiagen, Germantown, MD, USA). Total RNA (1 µg) from each sample was reverse transcribed using the iScript cDNA Synthesis Kit (Bio-Rad, Hercules, CA, USA). Quantitative real-time PCR was performed with the CFX 96TM Real-Time PCR System (Bio-Rad). Primer sequences are provided in Supplementary Table S1. Relative mRNA levels were determined using the cycle threshold (Ct) values, normalized to GAPDH, and compared to control samples as previously described [37]. Group differences were analyzed by Student’s *t*-test.

## 3. Results

### 3.1. ALDH2 KO Induces Proliferative Mammary Glands

**Figure 1:**
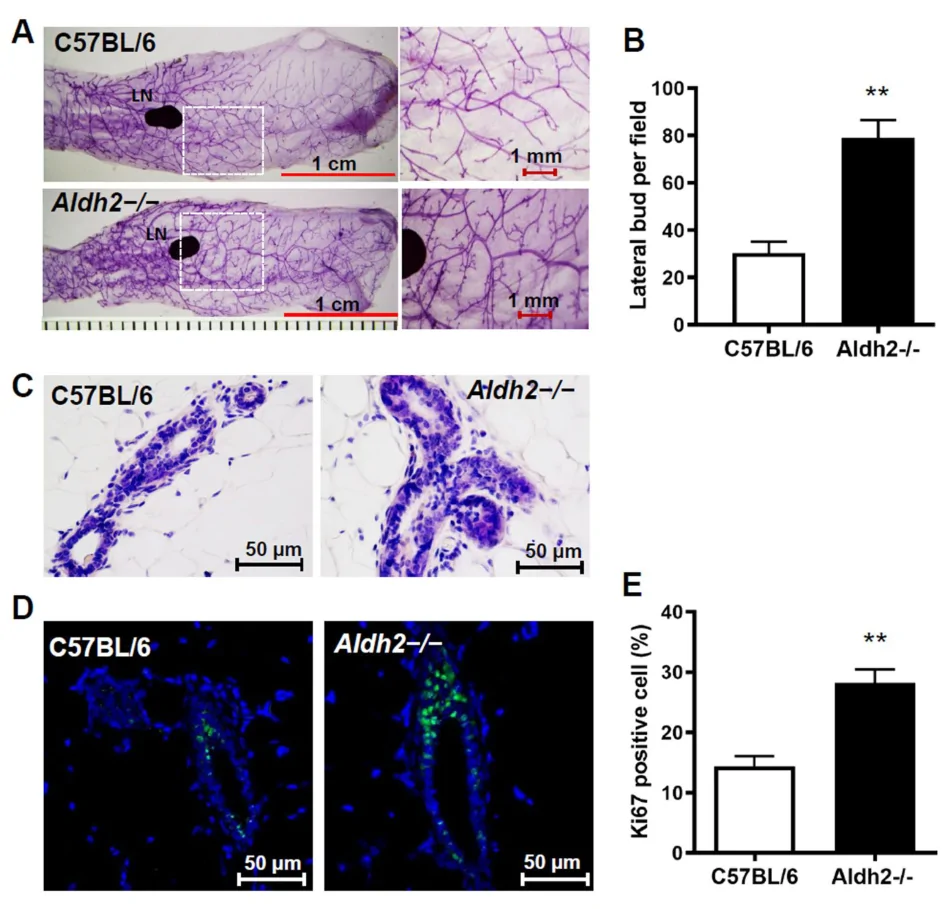
ALDH2 KO induces proliferative mammary glands in C57BL/6 mice. Mammary glands were collected for whole-mount and histopathology analyses at 16 weeks of age. (**A**) Representative mammary whole-mount images of control and Aldh2−/− mice. (**B**) Quantification of lateral buds per 10 mm^2^ (*n* = 5 mice per group). (**C**) H&E staining of mammary tissues showing ductal structures. (**D**) Ki67 immunofluorescence of mammary tissues from control and Aldh2−/− mice, with Ki67+ cells shown in green and nuclei stained with DAPI (blue). (**E**) Quantification of Ki67+ mammary epithelial cells based on 5 mice per group. Data are presented as mean ± SD (** *p* < 0.01).

The effect of ALDH2 deficiency on mammary development has not been previously reported. To investigate the impact of ALDH2 KO on mammary gland development and morphogenesis, we performed histological analyses of tissues from control and Aldh2−/− mice. Based on our initial characterization across different ages, we selected 16 weeks of age for subsequent analyses because morphological differences between control and Aldh2−/− mice were consistently evident at this stage. Whole-mount analysis revealed more complex ductal growth in Aldh2−/− mice (Figure 1A), with increased lateral branching compared to the control (Figure 1B). H&E staining demonstrated increased ductal epithelial thickness with prominent epithelial multilayering/pseudostratification in the mammary ducts of Aldh2−/− mice compared with controls, consistent with a hyperplastic and proliferative epithelial phenotype (Figure 1C). Morphometric analysis further indicated that ALDH2 deficiency promotes mammary epithelial proliferation, which was supported by Ki67 immunostaining of mammary tissues (Figure 1D). Quantification revealed a significantly higher percentage of Ki67-positive cells in Aldh2−/− mice than in control mice (Figure 1E). Similar morphological differences persisted in aged animals, as whole-mount analysis of 73-week-old mice showed greater ductal complexity and epithelial density in Aldh2−/− mammary glands compared with age-matched controls (Supplementary Figure S1). Collectively, these findings demonstrate that ALDH2 deficiency promotes mammary epithelial proliferation and enhances mammary gland morphogenesis.

### 3.2. ALDH2 KO Induces Expansion of Both Luminal and Basal Mammary Epithelial Cell Subpopulations and Increases Luminal Progenitor Cells

**Figure 2:**
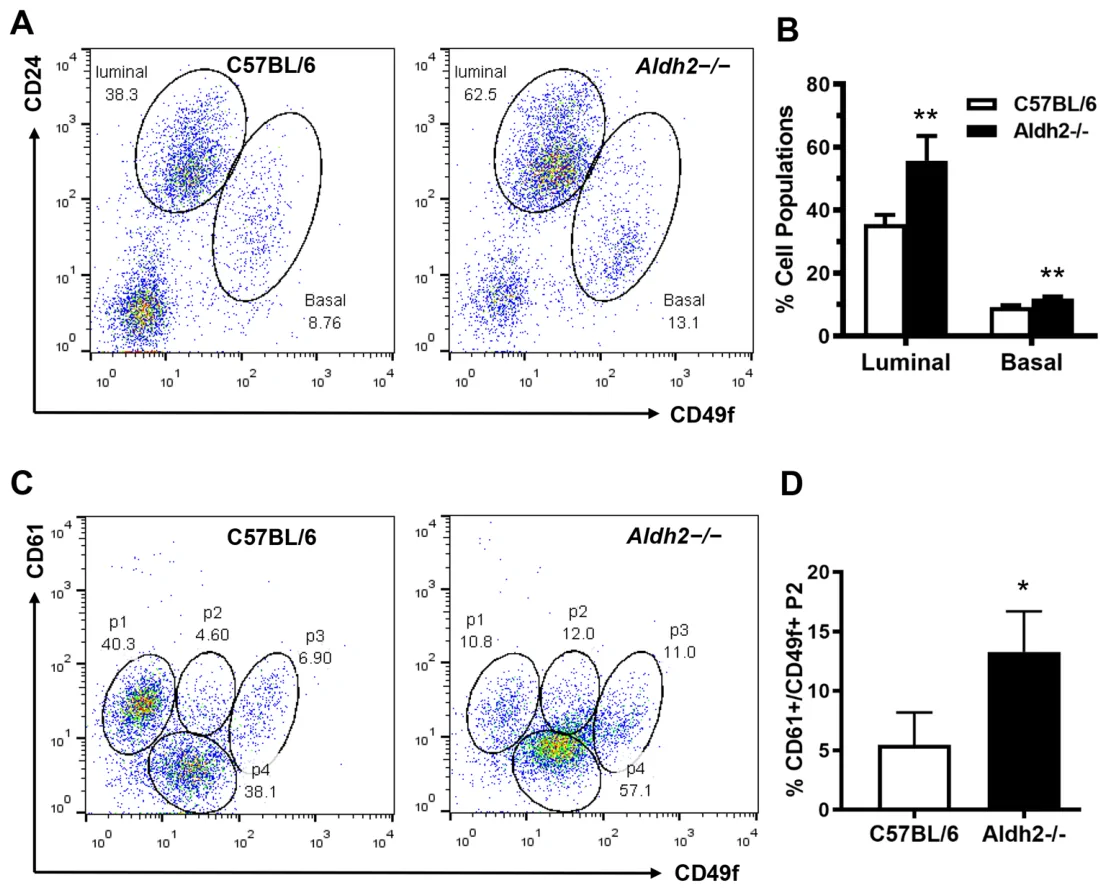
ALDH2 KO induces expansion of luminal and basal mammary epithelial cell subpopulations with increased luminal progenitor cells. Mammary epithelial cells from control and Aldh2−/− mice were analyzed for different mammary epithelial cell subpopulations by flow cytometry. CD24 and CD49f were used to detect the relative composition of luminal, basal, and stromal subpopulations (**A**,**B**). CD61 and CD49f were used to detect enriched subpopulations of luminal progenitor cells (CD61+/CD49f+) (**C**,**D**). (**A**) Representative plots based on CD24 and CD49f expression for luminal and basal subpopulations. (**B**) Percentages of luminal/basal subpopulations (*n* = 4 per group). (**C**) Representative plots showing subpopulation 2 (P2) of CD61+/CD49f+ cells in mammary tissues of control and Aldh2−/− mice. (**D**) Quantification of CD61+/CD49f+ P2 cells (*n* = 4 per group) (** *p* < 0.01, * *p* < 0.05).

Flow cytometric analysis of mammary epithelial cell (MEC) subpopulations is commonly used to characterize mammary epithelial cell composition, differentiation, and stem/progenitor cell populations [38,39]. To investigate the cellular mechanism underlying ALDH2 KO-induced mammary epithelial proliferation, we analyzed the relative composition of luminal, basal, and stromal subpopulations using CD24 and CD49f-based markers. Aldh2−/− mice exhibited significantly higher percentages of both basal and luminal cells compared to the control, suggesting that ALDH2 deficiency promotes mammary gland developmental activity and physiological remodeling (Figure 2A,B). CD61 is a recognized marker for luminal progenitor cells [40,41]. In our CD61/CD49f-based analysis of mammary epithelial cells, the P2 subpopulation represents enrichment of luminal progenitor cells (Figure 2C), which was significantly increased in the mammary tissues of Aldh2−/− mice. Our results indicate that the relative composition of luminal progenitor cells was significantly higher in Aldh2−/− mice compared to controls (Figure 2D). Collectively, flow cytometry data indicate that ALDH2 KO promotes expansion of stem/progenitor-enriched mammary epithelial populations, reflecting remodeling of the epithelial hierarchy and enhanced repopulation potential in Aldh2−/− mice. These findings indicate enhanced mammary epithelial repopulation potential associated with ALDH2 deficiency.

### 3.3. ALDH2 KO Promotes Mammary Epithelial Cell Stemness

**Figure 3:**
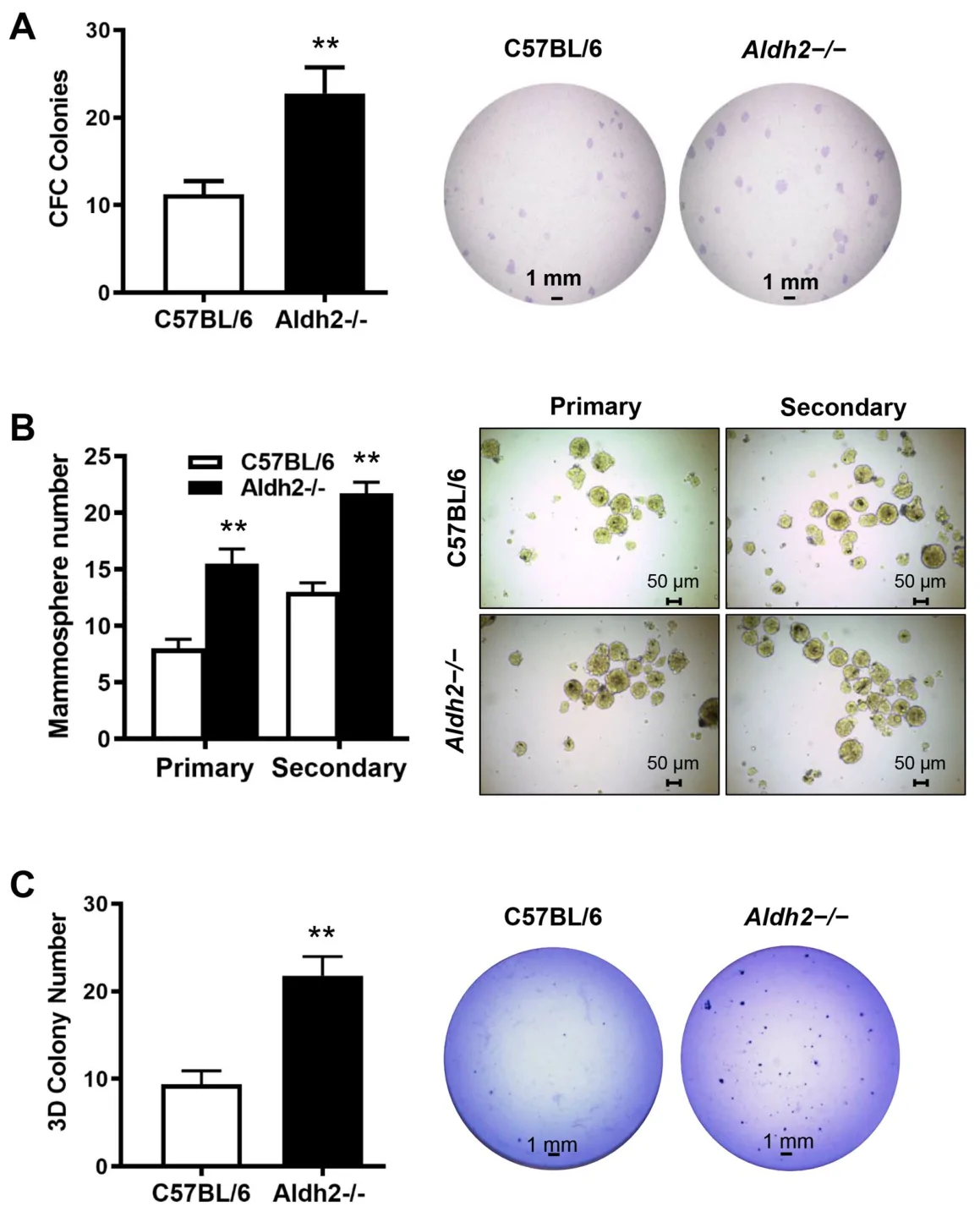
ALDH2 KO promotes mammary epithelial cell stemness. Primary MECs from control and Aldh2−/− mice (*n* = 5 mice per group) were evaluated in functional stemness assays. (**A**) Detection of relative colony-forming cell (CFC) numbers in the mammary tissues of control and Aldh2−/− mice with CFC assays. MECs from each group (4 × 10^3^ per dish) were plated, resulting colonies were counted and quantified. (**B**) Mammosphere formation efficiency of mammary epithelial cells from control and Aldh2−/− mice. Primary MECs were cultured in supplemented Epicult-B medium for 7 days to evaluate primary mammosphere formation. Single cells from the primary spheres were cultured under the same conditions to assess secondary mammospheres. Spheres > 30 μm were counted and quantified for statistical analysis. (**C**) 3D colony formation of MECs was assessed with a 3D culture assay. Primary MECs from each group were cultured in Matrigel for 10 days, followed by fixation, staining, and colony analysis. All assays were performed in triplicate (** *p* < 0.01).

The ALDH2 KO-associated expansion of basal, luminal, and progenitor-like cell subpopulations in Aldh2−/− mice, suggests that ALDH2 deficiency induces dynamic changes in mammary tissue stemness. To assess functional impact on mammary epithelial cell stemness, we performed colony-forming cell (CFC) assays, mammosphere formation, and 3D culture. The number of CFCs, a functional measure of luminal progenitor cell enrichment [42], was significantly increased in mammary tissues from Aldh2−/− mice compared with controls (Figure 3A). Mammosphere formation assays, which measure the self-renewal potential of mammary stem cells [42], demonstrated that both primary and secondary mammospheres derived from Aldh2−/− mice had significantly higher formation efficiency compared to controls (Figure 3B). Additionally, we performed 3D culture assays to assess the colony formation efficiency of mammary epithelial cells from both groups under semi-solid culture conditions. As shown in Figure 3C, the number of colonies in the Aldh2−/− group was significantly greater than in the control. Together, these findings indicate that ALDH2 deficiency promotes a stem/progenitor-enriched mammary epithelial state characterized by enhanced repopulation capacity, mammosphere formation, and colony-forming activity.

### 3.4. ALDH2 KO Activates Oxidative Stress Signaling via p38 MAPK and NF-κB and Induces DNA Damage in Mammary Tissues

**Figure 4:**
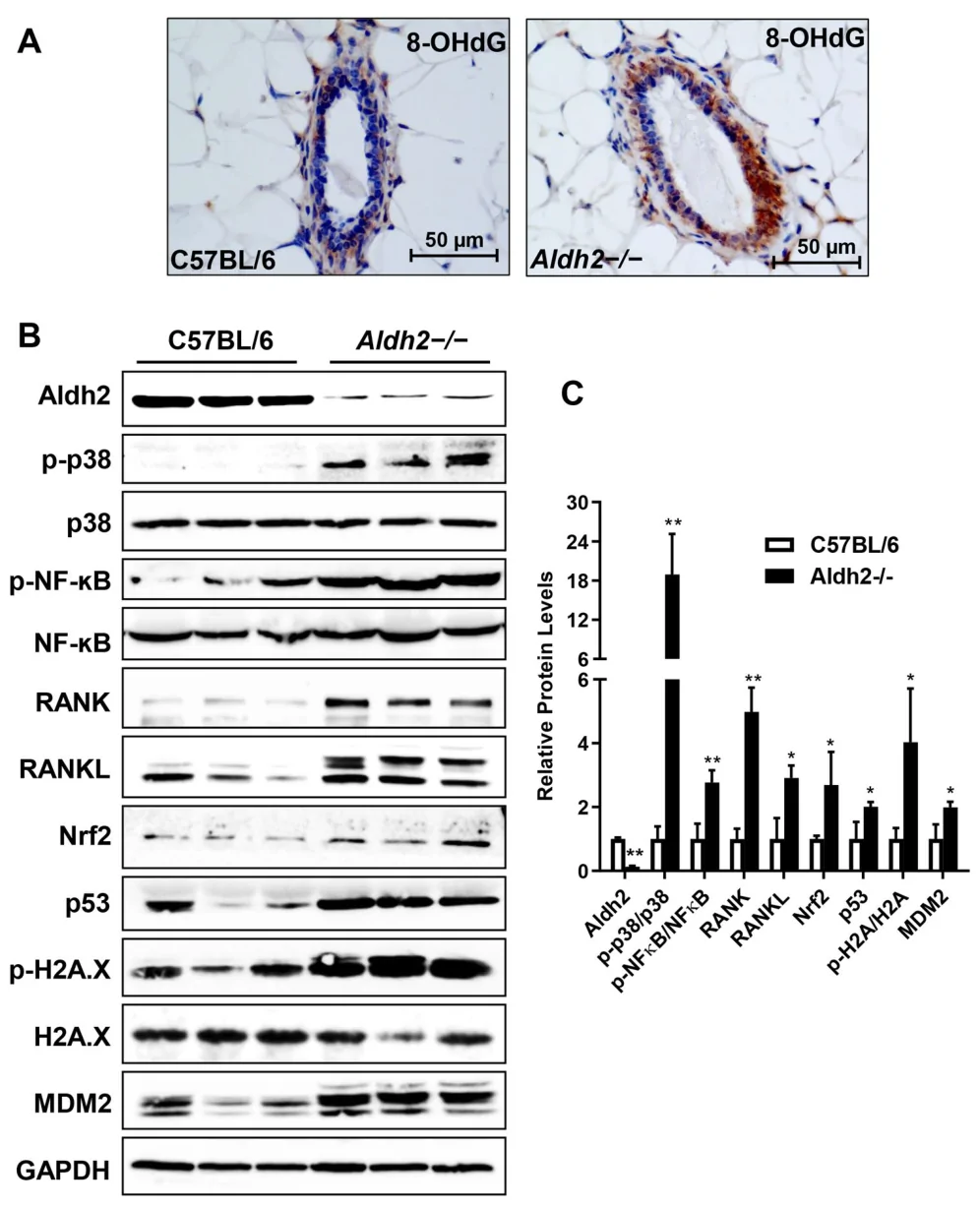
ALDH2 KO activates p38 MAPK- and NF-κB-associated oxidative stress and induces DNA damage in mammary tissues. (**A**) Immunohistochemical staining of 8-OHdG (brown) in mammary tissues of control and Aldh2−/− mice. (**B**) Western blot analysis showing ALDH2 KO-induced expression and phosphorylation of key markers of oxidative stress and DNA damage pathways in mammary tissues of control and Aldh2−/− mice. Protein levels of the indicated markers were assessed in three mice per group. (**C**) Densitometric quantification of Western blot bands shown in (**B**). Band intensities were normalized to the corresponding loading controls, with phosphorylated proteins further normalized to their respective total protein levels. Data represent the mean of three biological samples per group. * *p* < 0.05; ** *p* < 0.01.

Previous studies have shown that ALDH2 KO leads to aldehyde accumulation and increased oxidative stress, activating stress pathways in multiple tissues [21]. To investigate the molecular mechanisms underlying altered mammary development in Aldh2−/− mice, we examined oxidative stress markers in mammary tissues. Immunohistochemistry (IHC) of 8-OHdG, a widely used oxidative stress marker, revealed significantly increased 8-OHdG levels in the mammary epithelial cells of Aldh2−/− mice (Figure 4A), confirming oxidative stress induced by ALDH2 deficiency. Western blotting demonstrated alterations in key oxidative stress pathway markers, showing that protein levels of phosphorylated p38 MAPK and NF-κB were significantly upregulated in the mammary tissues of Aldh2−/− mice relative to controls (Figure 4B,C). These changes were accompanied by increased Nrf2 expression, a master regulator of antioxidant response [43]. This underscores the role of p38 MAPK, NF-κB, and Nrf2 activation in the interplay between oxidative stress signaling, inflammation, and antioxidant defense in ALDH2-deficient mammary tissues. To further explore NF-κB signaling, we examined whether ALDH2 knockout modulates expression of receptor activator of nuclear factor κB ligand (RANKL), a regulator of osteoclast differentiation increasingly implicated in mammary stem cell regulation and breast oncogenesis [44]. Immunoblotting revealed increased expression of full-length RANKL and an additional higher-molecular-weight band in Aldh2−/− mammary tissues, suggesting that ALDH2 deficiency enhances overall RANKL expression as well as the abundance of alternative isoforms or processed forms (Figure 4B,C). These findings suggest an association between RANKL upregulation, stress signaling, and enhanced mammary epithelial stemness. To assess whether increased oxidative stress is associated with DNA damage, we examined DNA damage signaling in ALDH2 KO mammary tissues. Protein levels of DNA damage markers, p53 and phosphorylated H2A.X, were markedly increased in Aldh2−/− tissues. MDM2, an oncogenic E3 ubiquitin ligase regulated by p53, was also significantly upregulated. Of note, as these samples were derived from individual mice, inter-animal variability in protein expression was observed for certain markers (e.g., p53 and MDM2); therefore, the overall changes were evaluated based on the expression patterns together with densitometric analysis of individual samples. Collectively, these results demonstrate that ALDH2 deficiency induces a robust oxidative stress response accompanied by activation of DNA damage signaling pathways in mammary tissues through activation of p38 MAPK, NF-κB, and Nrf2 pathways.

### 3.5. ALDH2 KO Induces Hormonal Signaling Dysregulation and Concomitant Activation of erbB3-Associated Signaling in Mammary Tissues

**Figure 5:**
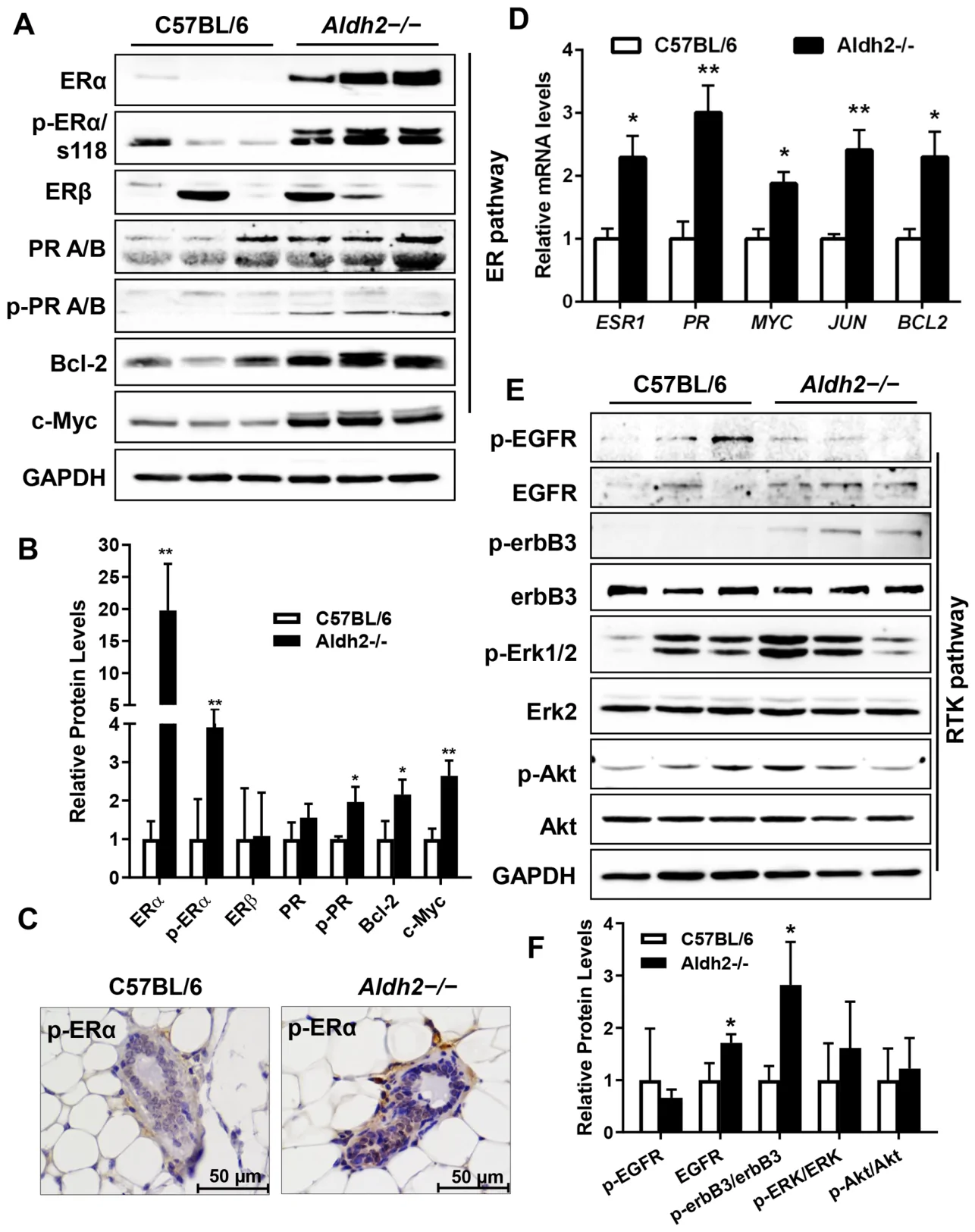
ALDH2 KO upregulates estrogen receptor signaling in coordination with receptor tyrosine kinase pathways in mammary tissues. (**A**) Western blot analysis of ER, PR, and selected target proteins in mammary tissues from three mice per group. (**B**) Densitometric quantification of Western blot bands shown in (**A**). Band intensities were normalized to the corresponding loading controls, with phosphorylated proteins further normalized to their respective total protein levels. Data represent the mean of three biological samples per group. (**C**) IHC staining of phosphorylated ERα (S118) (brown) in control and Aldh2−/− mammary tissues. Scale bars, 50 μm. (**D**) Quantitative real-time PCR analysis of relative mRNA levels of selected ER target genes. Relative mRNA levels were normalized to GAPDH. (**E**) Western blot analysis of EGFR/ErbB-associated signaling markers in control and Aldh2−/− mammary tissues. (**F**) Densitometric quantification of Western blot bands shown in (**E**). Total EGFR and p-EGFR were independently normalized to the loading control, whereas p-ErbB3, p-ERK1/2, and p-Akt were normalized to their corresponding total protein levels. Data represent the mean of three biological samples per group. * *p* < 0.05; ** *p* < 0.01.

Hormonal signaling is central to mammary development and oncogenesis [45]. To investigate the impact of ALDH2 KO on ER/PR signaling, we performed Western blot analysis, which revealed significantly increased protein levels of ERα, PR, and phosphorylated ERα (Ser118) (Figure 5A,B), suggesting that ALDH2 KO induces both the expression and phosphorylation/activation of ER signaling. Consistent with this, ER target proteins, including c-Myc and Bcl-2, were also upregulated. These results indicate that ALDH2 deficiency strongly enhances ER signaling. To validate this finding, we examined in situ signals of phosphorylated ERα (S118) in mammary tissues by IHC. pERα staining was predominantly localized to the nuclei of mammary epithelial cells and was increased in Aldh2−/− tissues (Figure 5C). Additionally, to further demonstrate ALDH2 KO-induced activation of ER signaling, we measured mRNA levels of several ER-target genes, including *ESR1/ERα*, *PR*, *MYC*, *JUN*, and *BCL2*. The results revealed that the mRNA levels of these ER-target genes in the mammary tissues of Aldh2−/− mice were significantly upregulated (Figure 5D). Collectively, these results provide strong in vivo evidence that ALDH2 deficiency induces ERα expression and activation, potentially contributing to proliferative mammary development and epithelial cell repopulation.

Given the crosstalk between ER and RTK signaling [46], we examined the protein levels of EGFR, ErbB3, and downstream markers of ERK and Akt in the mammary tissues of both groups. ErbB3 activation and phosphor-EGFR levels were significantly increased in Aldh2−/− mammary tissues, accompanied by trends toward increased EGFR, p-ERK1/2, and p-Akt levels, although these changes did not reach statistical significance due to inter-animal variability (Figure 5E,F). These findings suggest coordinated alterations and a trend toward enhanced ER/RTK-associated signaling in ALDH2-deficient mammary tissues.

### 3.6. ALDH2 KO Induces Marked Upregulation of Cyclin D1 and the Activation of Rb-E2F1 Pathway in Mammary Tissues

**Figure 6:**
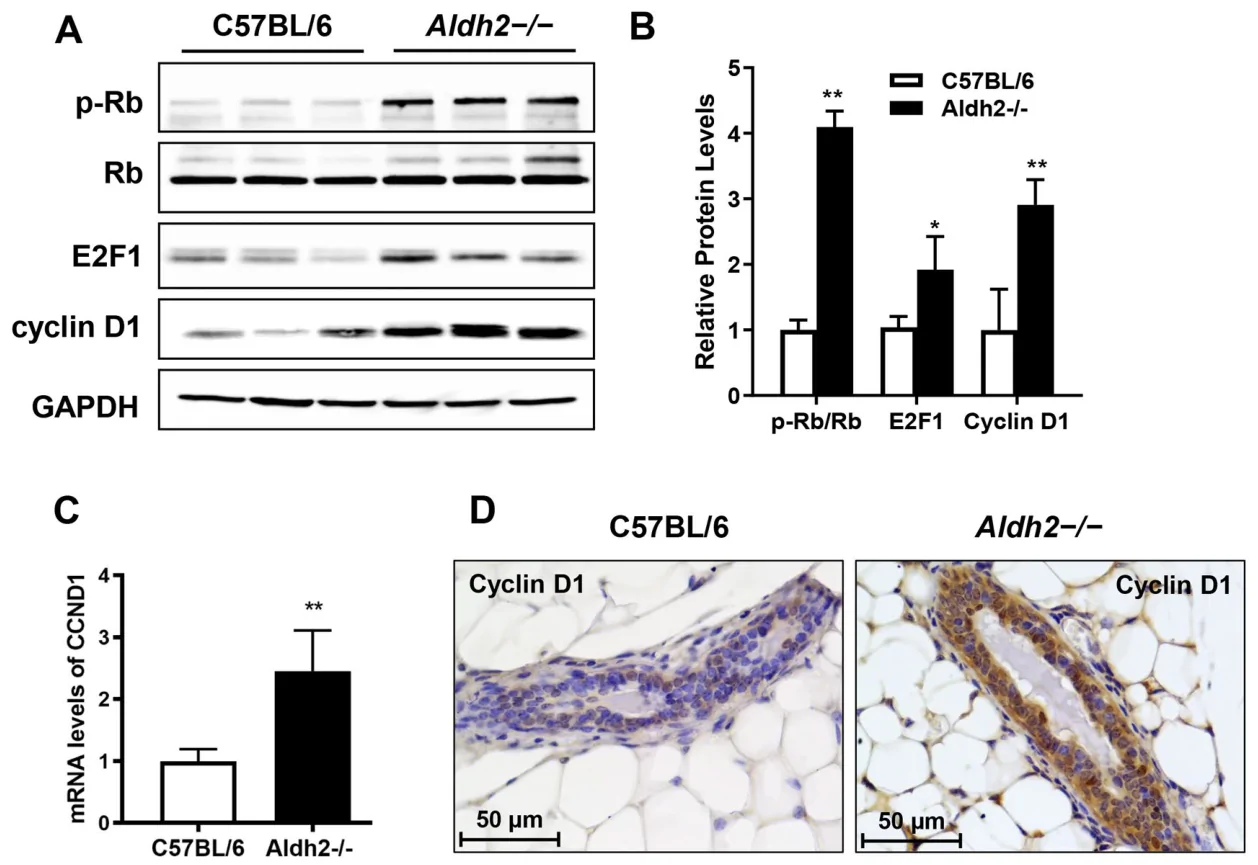
ALDH2 KO induces cyclin D1 upregulation and activates the Rb–E2F1 pathway in mammary tissues. (**A**) Western blot analysis of Rb, E2F1, and cyclin D1 in mammary tissues of control and Aldh2−/− mice, probed with specific antibodies as described in Figure 4. (**B**) Densitometric quantification of Western blot bands shown in (**A**). Band intensities were normalized to the corresponding loading controls, with phosphorylated Rb further normalized to total Rb. Data represent the mean of three biological samples per group. (**C**) Relative mRNA levels of CCND1 (cyclin D1) quantified by real-time PCR as described in Figure 5. (**D**) Immunohistochemical staining of cyclin D1 (brown) in mammary tissues from both groups. Scale bars, 50 μm. * *p* < 0.05; ** *p* < 0.01.

The Rb/E2F1–cyclin D1 axis is a central regulator of cell cycle progression and proliferation [46]. To investigate ALDH2 KO-induced mammary epithelial proliferation, we analyzed the expression of cell cycle markers, including Rb, E2F1, and cyclin D1. ALDH2 KO significantly increased Rb phosphorylation and upregulated E2F1 protein levels (Figure 6A,B). Notably, cyclin D1 protein levels were markedly increased, which was corroborated by increased CCND1 mRNA levels (Figure 6C) and enhanced IHC staining of cyclin D1 in the mammary tissues (Figure 6D). Collectively, these findings highlight cell cycle progression as a major pathway affected by ALDH2 KO. As cyclin D1 is a downstream target of ER signaling, its pronounced upregulation validates ER pathway activation in Aldh2−/− tissues. This result further suggests that cyclin D1 emerges as a prominent downstream effector of ALDH2 deficiency-associated ER signaling and a useful marker of proliferative mammary remodeling.

## 4. Discussion

The present study provides novel insights into the impact of ALDH2 deficiency on mammary gland development and the underlying cellular mechanisms involving alterations in cell proliferation, stemness, and associated signaling pathways. As the first study to investigate ALDH2 deficiency-associated mammary pathophysiology, our findings demonstrate that ALDH2 KO enhances oxidative stress and induces significant structural and functional alterations in mammary tissues at the molecular level. Notably, the association of ALDH2 KO-induced ER signaling and proliferation with alterations in epithelial cell stemness could be pertinent for understanding breast cancer pathogenesis.

Our histopathological analysis indicates that ALDH2 KO leads to more complex ductal growth and increased ductal thickness in mammary glands compared with controls, as demonstrated by whole-mount and H&E staining (Figure 1). This observation was further supported by Ki67 staining, which revealed a higher percentage of proliferative cells in Aldh2−/− tissues. Notably, similar morphological differences persisted in aged animals, as whole-mount analysis of 73-week-old mice showed greater ductal complexity and significantly increased lateral budding in Aldh2−/− mammary glands compared with age-matched controls (Supplementary Figure S1), suggesting that the mammary phenotype associated with ALDH2 deficiency persists with age. The implication of enhanced mammary gland proliferation aligns with previous findings showing that alterations in metabolic enzymes can impact cell growth and tissue remodeling in other tissues [47]. The accumulation of endogenous toxic aldehydes or other metabolites due to ALDH2 deficiency may exacerbate oxidative stress, thereby contributing to hyperplasia and increased cellular turnover in mammary tissues.

Our exploration of the cellular mechanisms revealed an expansion of both luminal and basal epithelial cell populations in Aldh2−/− mice, as indicated by CD24/CD49f-based flow cytometry analysis (Figure 2). Basal subpopulation, which typically includes stem and progenitor cells that play a crucial role in mammary development and tumorigenesis [48], may suggest enhanced stem cell activity and mammary epithelial cell proliferation. In parallel, the luminal subpopulations, composed of differentiated cells that line the inner layer of mammary ducts and alveoli [49], were also elevated, indicating enhanced differentiation or glandular activity. Notably, CD61/CD49f analysis demonstrated a significant increase in the progenitor-enriched P2 subpopulation (Figure 2C), consistent with the established role of luminal progenitor cells in normal mammary gland development and tumorigenesis [40,41]. The observed epithelial cell repopulation and enrichment of potential progenitor cells suggest that ALDH2 deficiency influences not only the overall hierarchy of mammary epithelial cells but also the stemness of these cells, potentially expanding the pool of cells that could give rise to tumor initiation cells in the presence of other factors [35]. The above observed mammary epithelial cell repopulation is also supported by our assessment of mammary epithelial cell stemness in the Aldh2−/− model using colony-forming assays and mammosphere formation studies (Figure 3). The increased colony numbers and enhanced mammosphere formation efficiency indicate that ALDH2 deficiency promotes stem cell characteristics among mammary epithelial cells. These novel findings underscore the potential contribution of ALDH2 deficiency-altered metabolic states and microenvironment to modified stem cell dynamics that may promote the emergence of tumor initiation cells. Taken together, the alterations in the intrinsic regulation of mammary epithelial cells in this model system, along with the methodologies employed in this study, offer a solid basis and valuable resources for investigating ALDH2 deficiency-induced mammary tumorigenesis using other mammary tumor models.

The development and function of the mammary gland, as well as the behavior of mammary epithelial cells, are highly sensitive to cellular stress and changes in the microenvironment [50]. Oxidative stress is one such factor that can profoundly affect mammary gland development and epithelial cell dynamics [51,52], with consequences that vary depending on stimulus and stress intensity. However, the impact of ALDH2 deficiency-associated oxidative stress on mammary development and epithelial cell regulation in the absence of exogenous alcohol exposure has not been documented. Consistent with the observed phenotypic changes, our analysis of oxidative stress signaling and DNA damage reveals molecular mechanisms by which Aldh2 KO alters mammary epithelial stemness. The upregulation of the p38 MAPK and NF-κB pathways in Aldh2−/− mammary tissues indicates strong activation of cellular stress responses (Figure 4), while induction of Nrf2 highlights activation of the antioxidant defense mechanism. Although the roles of oxidative stress markers such as p38 MAPK and NF-κB in cancer are well established [53,54], the specific impact of ALDH2 deficiency and the accumulation of endogenous toxic metabolites in mammary tissues and cells remains largely unexplored. In this regard, consistent with previous studies demonstrating endogenous aldehyde accumulation following ALDH2 deficiency [55], our findings show that ALDH2 loss is associated with increased oxidative stress and DNA damage in mammary tissues, as evidenced by increased 8-OHdG, p53, and H2A.X. This is also in accordance with recent studies indicating that aldehydes, whether alcohol-induced or endogenous, impair adult stem cells, including hematopoietic, neural, and intestinal stem cells [56,57,58]. To strengthen the association between ALDH2 deficiency, endogenous aldehyde accumulation, and the observed phenotypic changes, future studies will incorporate direct aldehyde measurements and functional rescue approaches to establish the causal relationships.

Our findings identify RANKL induction as a previously unrecognized consequence of ALDH2 deficiency and suggest a potential mechanism linking aldehyde stress to mammary epithelial stemness and proliferative remodeling. Our study also demonstrates for the first time that ALDH2 deficiency markedly upregulates RANK and RANKL in mammary tissues, consistent with the established involvement of RANKL in tumorigenesis [59]. While RANKL expression is known to be induced by hormonal signals such as progesterone and hypoxia [59,60], its regulation by metabolic enzyme deficiency has not been reported. Given RANKL’s established role in mammary stem cell regulation and its ability to act upstream of NF-κB and p38 MAPK signaling [59,61], this upregulation may amplify stress signaling and stem-like features in breast cancer cells [62]. Collectively, our findings support a hypothetical model in which ALDH2 deficiency-associated metabolic and oxidative stress is accompanied by coordinated dysregulation of RANK/RANKL, ER/PR, and RTK/ErbB3 signaling, contributing to altered mammary epithelial homeostasis. Among these pathways, RANK/RANKL induction represents a particularly novel finding and may provide an important link between aldehyde-associated stress and mammary epithelial proliferation and stemness. However, the present data are correlative, and the causal contribution and hierarchy of these signaling pathways remain to be established through pathway-specific inhibition or genetic perturbation.

ER signaling is essential for normal mammary gland development and plays a significant role in breast cancer risk [63,64,65]. Our results indicate that ALDH2 deficiency significantly activates ER signaling pathway in mammary tissues, evidenced by elevated ERα and PR levels, together with increased phosphorylation of ERα (S118) and PR (S190) in mammary tissues with ALDH2 KO (Figure 5). The upregulation of ER target genes, such as Bcl-2, cyclin D1, and c-Myc, along with IHC detection of p-ERα (S118), further supports this activation. Notably, the pronounced increase in cyclin D1 in ALDH2 KO tissues may contribute to enhanced Rb/E2F1-cyclin D1 axis signaling (Figure 6). Given the central role of ER/PR signaling in mammary epithelial proliferation and the maintenance of stemness [63], our results not only provide in vivo evidence of ALDH2 KO-mediated upregulation of ER signaling but also suggest a mechanistic role for ER/PR signaling dysregulation in ALDH2 deficiency-induced mammary epithelial cell proliferation and stemness. In addition, ALDH2 KO induced phosphorylation and activation of erbB3 and downstream signaling (Figure 5). ErbB3, a potent receptor tyrosine kinase (RTK) of the EGFR/erbB family, is known to regulate mammary development and tumorigenesis [66]. Among the erbB family members, the ErbB2/ErbB3 axis is strongly associated with breast tumor progression, whereas the ErbB2/ErbB4 axis plays important roles in normal mammary development [67,68]. The distinct upregulation of erbB3 in Aldh2−/− tissues suggests its involvement in ALDH2-related epithelial cell proliferation and repopulation. In the present study, ErbB2 expression was too low for reliable evaluation, and ErbB4 was not examined. Further studies are needed to determine whether ALDH2 deficiency alters these additional ErbB signaling pathways. Ongoing studies are investigating the impact of ALDH2 deficiency on ErbB2-driven mammary tumorigenesis. Since ERα-S118 is a substrate of Erk1/2 [69], the observed increases in Erk1/2 activation and phosphorylated ERα-S118 emphasize the connection between ER and RTK pathways in ALDH2 deficiency-induced mammary alterations. Notably, activation of both ER signaling and erbB3 in the context of ALDH2 deficiency has not been reported previously, warranting further investigation. In the context of breast oncogenesis, our findings suggest that dysregulation of hormonal signaling by ALDH2 deficiency may create a mammary epithelial state with increased susceptibility to oncogenic transformation.

In summary, our findings provide strong evidence that ALDH2 deficiency promotes mammary epithelial cell proliferation and morphogenesis, accompanied by altered stem-like properties. By elucidating the role of ALDH2 in maintaining epithelial cell integrity and regulating stemness, we offer new insights into mammary gland development and its potential implications for breast cancer biology. Notably, these changes were observed in the Aldh2−/− model in the absence of exogenous alcohol exposure. These findings raise the possibility that impaired metabolism of endogenous aldehydes contributes to the altered mammary phenotype associated with ALDH2 deficiency. Furthermore, our study highlights how metabolic dysregulation, particularly oxidative stress associated with ALDH2 deficiency, is accompanied by changes in mammary tissue remodeling, cellular proliferation, and stemness. This model system provides a foundation for investigating ALDH2 deficiency-induced intrinsic metabolic and signaling pathways and their potential contribution to breast cancer development. Given the established role of ALDH2 in detoxifying endogenous reactive aldehydes, we hypothesize that impaired clearance of endogenous aldehydes may contribute to the increased oxidative stress observed in ALDH2-deficient mammary tissue. Key questions remain, including whether ALDH2 deficiency enhances alcohol-induced mammary epithelial proliferation and stemness, and whether it promotes tumorigenesis in mammary tumor models. These questions are being addressed in our ongoing projects. Importantly, the potential relevance of ALDH2 deficiency to breast cancer is not limited to the ALDH2*2 polymorphism. Previous reports based on clinical samples and public cancer datasets, including TCGA, have reported reduced ALDH2 expression in breast tumors compared with normal breast tissues and associations between lower ALDH2 expression and poorer clinical outcomes [70,71]. These clinical observations suggest an association between altered ALDH2 expression and breast cancer biology, but do not establish reduced ALDH2 expression as a determinant of breast cancer susceptibility. Nevertheless, the Aldh2−/− model represents complete loss of ALDH2 expression and is mechanistically distinct from the human ALDH2*2 polymorphism, which produces a dominant-negative variant protein; therefore, the knockout model should be considered a proof-of-concept model for defining the biological consequences of complete ALDH2 loss rather than a direct model of the human ALDH2*2 genotype. Future validation using tumor models and human breast tissue samples would further strengthen the clinical and translational relevance of these findings. In considering the implications of our findings for tumor tissues, ALDH2 loss in normal mammary epithelium alters oxidative stress, proliferation, morphogenesis, and stem/progenitor-associated properties. These effects may be further modified or intensified in established tumors in the context of tumor-associated metabolic and signaling dysregulation. Further studies are needed to define the context-dependent role of ALDH2 in mammary tumor cells.

While the observed changes in epithelial subpopulations, progenitor enrichment, and stem-like properties indicate substantial alterations in mammary epithelial cell dynamics, their functional consequences for mammary differentiation and lactation remain to be determined. Similarly, the proliferative and stem-like phenotypes observed here may suggest increased susceptibility to oncogenic transformation but do not establish an effect on tumorigenesis. Future studies will be needed to define these physiological consequences, such as lactation. The effect of ALDH2 deficiency on mammary tumor development is being investigated in ongoing studies.

In conclusion, our investigation highlights the multifaceted impact of ALDH2 deficiency on mammary gland development, including alterations in epithelial cell proliferation and stemness. Alterations in cell proliferation, stemness, and oxidative stress responses, hormonal modulation, and RANK/RANKL signaling pathways highlight ALDH2 as a central regulator of mammary biology. As the interplay between genetic and metabolic factors in breast cancer becomes increasingly complex, further research into the molecular mechanisms of ALDH2 deficiency and its potential implications for breast cancer biology will be critical for developing targeted prevention strategies in susceptible populations. By elucidating how ALDH2 influences mammary development, future studies may also identify therapeutic targets for breast cancer intervention.

## Data Availability

The original contributions presented in this study are included in the article/Supplementary Material. Further inquiries can be directed to the corresponding author.

## Supplementary Materials

The following supporting information can be downloaded at https://www.mdpi.com/article/10.3390/cells15181632/s1. Figure S1: ALDH2 KO induces proliferative mammary glands in C57BL/6 mice; Table S1: Primer sequences used for qRT-PCR.
