# Supplementary material for: ALDH2 Deficiency Promotes Mammary Epithelial Stemness and Proliferative Morphogenesis Through Oxidative Stress, RANKL Induction, and Estrogen Receptor Signaling
Source: Cells. 2026 Sep 9;15(18):1632. doi: 10.3390/cells15181632 (PMC13605752; doi:10.3390/cells15181632)
Supplement: Supplementary file 1 [file cells-15-01632-s001.zip › cells-4391762-supplementary.pdf]

Supplementary 1

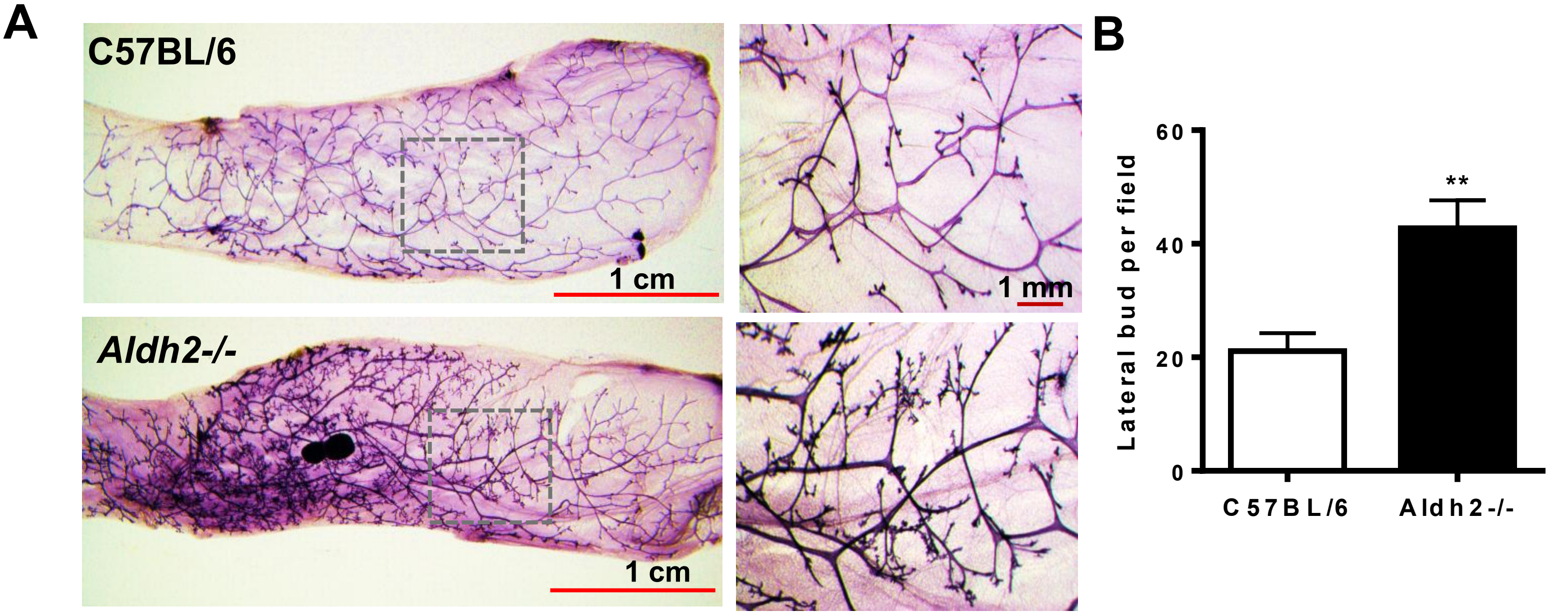

Figure S1. ALDH2 KO induces proliferative mammary glands in C57BL/6 mice. Mammary glands were collected for whole-mount and histopathology analyses at 73 weeks of age. A) Representative mammary whole-mount images of control and Aldh2-/- mice. B) Quantification of lateral buds per field (n = 5 mice per group).

Supplementary Table S1. Primer sequences used for qRT-PCR.

Primer Name	Sequence
ESR1	**F:** 5'-TCTCTGG AAGAGAAGGACCACATC-3'<br>**R**: 5'-TGCAGAGTCAGGCCAGCTTT-3'
JUN	**F:** 5'-AAAACCTTGAAAGCGCAAAA-3'<br>**R**: 5'-GTTTGCAACTGCTGCGTTAG-3'
MYC	**F:** 5'-TGAGCCCCTAGTGCTGCA-3'<br>**R**: 5'-AGCCCGACTCCGACCTCTT-3'
CCND1	**F:** 5'-GGGCACCTGGATTGTTCT-3'<br>**R**:5'-CACCGGAGACTCAGAGCA-3'
PR	**F:** 5'-CACAGCGCTTCTACCAACTCACAA-3'<br>**R**: 5'-TTGGGCAACTGGGCAGCAATAA-3'
BCL2	**F:** 5'-GGTCTTCAGAGACAGCCAGG-3'<br>**R**: 5'-GATCCAGGATAACGGAGGCT-3'
GAPDH	**F**: 5'-TGCGACTTCAACAGCAACTC-3<br>**R**: 5'-CTTGCTCAGTGTCCTTGCTG -3'
